# Epidemiological characterization of malaria in rural southern Tanzania following China-Tanzania pilot joint malaria control baseline survey

**DOI:** 10.1186/s12936-018-2446-7

**Published:** 2018-08-13

**Authors:** Rashid A. Khatib, Prosper P. Chaki, Duo-Quan Wang, Yeromin P. Mlacha, Michael G. Mihayo, Tegemeo Gavana, Ning Xiao, Xiao-Nong Zhou, Salim Abdullah

**Affiliations:** 1 Ifakara Health Institute, Kiko Avenue, Mikocheni, P.O. Box 78373, Dar es Salaam, United Republic of Tanzania; 2 Swiss Tropical and Public Health Institute (Swiss TPH), Socinstrasse 57, P.O. Box, 4002 Basel, Switzerland; 3 University of Basel, Petersplatz 1, 4003 Basel, Switzerland; 4 National Institute of Parasitic Diseases, Chinese Center for Disease Control and Prevention, 207 Rui Jin Er Road, Shanghai, 200025 People’s Republic of China

## Abstract

**Background:**

Malaria is an important public health problem in Tanzania. The latest national malaria data suggests rebound of the disease in the country. *Anopheles arabiensis*, a mosquito species renowned for its resilience against existing malaria vector control measures has now outnumbered the endophagic and anthrophilic *Anopheles gambiae* sensu stricto as the dominant vector. Vector control measures, prophylaxis and case management with artemisinin-based combination therapy (ACT) are the main control interventions. This paper presents and discusses the main findings from a baseline household survey that was conducted to determine malaria parasite prevalence and associated risk exposures prior to piloting the T3-initiative of World Health Organization integrated with Chinese malaria control experience aimed at additional reduction of malaria in the area.

**Methods:**

The study was conducted from 4 sub-district divisions in Rufiji District, southern Tanzania: Ikwiriri, Kibiti, Bungu, and Chumbi. Malaria transmission is endemic in the area. It involved 2000 households that were randomly selected from a list of all households that had been registered from the area. Residents in sampled households were interviewed on a range of questions that included use of long-lasting insecticidal nets (LLINs) the night prior to the interview and indicators of socio-economic status. Blood drops were also collected on blood slides that were examined for malaria parasites using microscopes.

**Results:**

The study observed an average malaria parasite prevalence of 13% across the selected site. Its distribution was 5.6, 12.8, 16.7, and 18% from Ikwiriri, Kibiti, Bungu, and Chumbi wards, respectively. The corresponding LLIN use discovered were 57.5% over the district. The highest usage was observed from Ikwiriri at 69.6% and the lowest from Bungu at 46.3%. A statistically significant variation in parasitaemia between socio-economic quintiles was observed from the study. Males were more parasitaemic than females (p value = 0.000).

**Discussion and conclusion:**

The findings have been discussed in the light of results from Tanzania Demographic and Health Survey-Malaria Indicator Survey, 2015–2016 and other related studies, together with goals and targets set for malaria control. The paper also discusses the observed parasitaemia in relation to reported LLIN use and its distribution by some important factors as they were explored from the study. It has been concluded that malaria burden is now concentrated on the fringes of the settlements where the poorest section of the population is concentrated and LLIN usage is lower than the national average and targets set by national and global malaria control initiatives.

## Background

Malaria is one of the communicable diseases accounting for major health burden in Tanzania. More than 90% of the population is at risk of transmission. Tanzania Demographic and Health Survey–Malaria Indicator Survey (TDHS–MIS) 2015–2016 for under-fives using malaria rapid diagnostic test (mRDT) shows average parasite prevalence of 14% [1]. This figure suggests that that the disease has rebound in the country as prevalence was less than 10% in 2012. The main vector species are *Anopheles gambiae* sensu stricto that has currently been outnumbered by *Anopheles arabiensis*, and *Anopheles funestus* is also active in some areas [2, 3]. The dominant parasite is *Plasmodium falciparum* [1]. The stated prevalence reported for the country is contrary to milestones set in the country’s national strategic plan for malaria that was 5% in 2016 and a decrease to less than 1% in 2020 [4]. The major control strategies in place are long-lasting insecticidal nets (LLINs), indoor residual spraying (IRS), larviciding of mosquito breeding sites, intermittent presumptive therapy in pregnant women (IPTp), quality-assured diagnostic testing and treatment of malaria cases with artemisinin-based combination therapy (ACT) [4]. Coverage level for all these interventions fell in 2016 against the progress that had been made in 2012 [1]. Against this background, the China-Tanzania joint malaria control project selected Rufiji district, Coast region in southern Tanzania for piloting the T3-initiative of the World Health Organization-integrated with Chinese malaria control experience aimed at additional reduction of malaria in the area.

The project conducted a baseline household survey across Rufiji district to determine some key metrics of malaria epidemiology in the area. Milestones and targets set by the project will be measured using these baseline parameters. This paper aims at shedding light on these baseline parameters in the area and they will be discussed in the light of the overall malaria condition in the country as highlighted from the TDHS–MIS 2015–2016 [1].

The study aimed at identifying the baseline malaria parasite burden and related risk factors in four wards of Rufiji District, Coast Region.

## Methods

### Study site

Rufiji is a district located in Coast region, Tanzania. It lies about 200 km south of Dar es Salaam extending between 7.470 and 8.030°S and 38.620 and 39.170°E along the Dar es Salaam-Lindi and Mtwara Highway. It occupies a land area of 14,500 sq km (divided into 19 wards and 100 villages), which is almost half of the land of the administrative region to which it belongs and bigger than the two smallest regions on Tanzania mainland. The district is named after the country’s largest river, Rufiji River, through which its longest stretch passes, and its huge valley and flood plain defines its ecology, settlement pattern and economic activities.

**Fig. 1:**
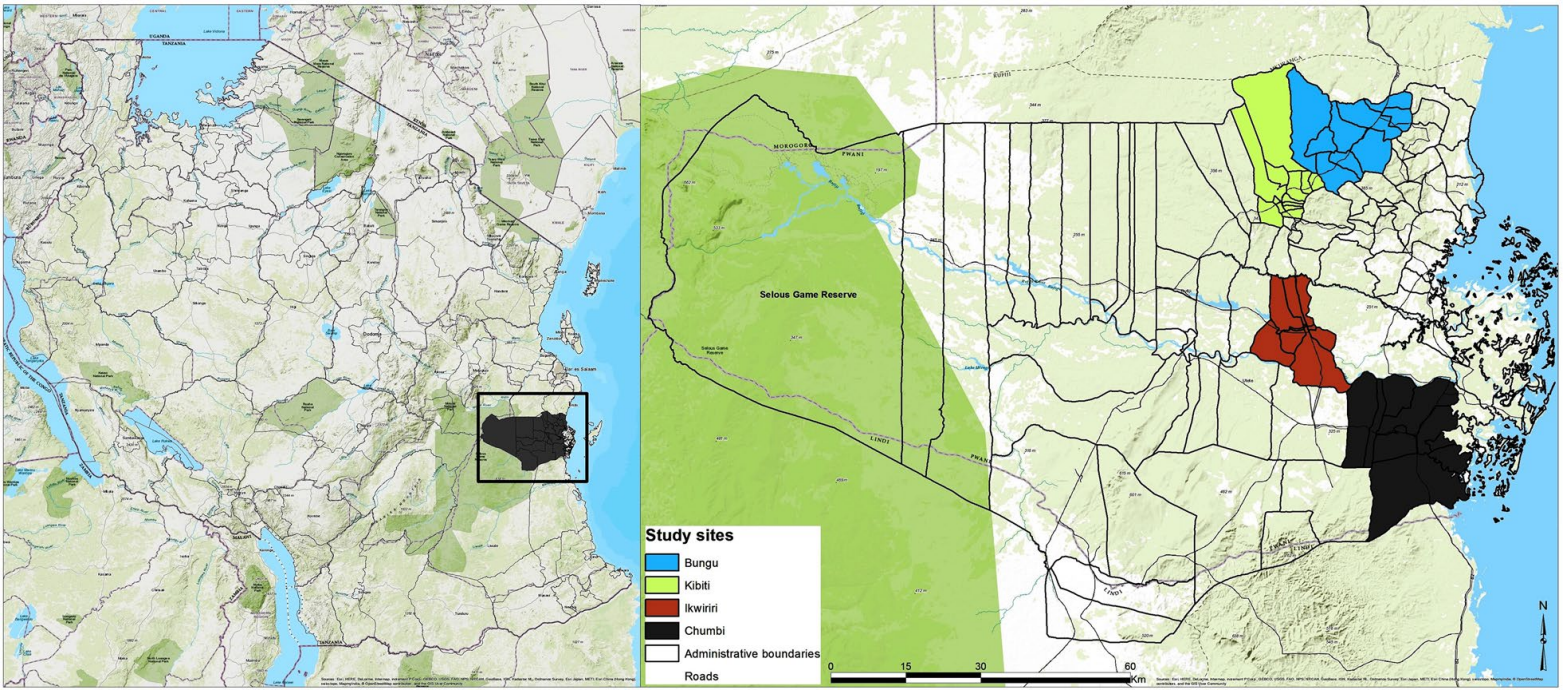
Study area location

The district’s current population is 248,230 scattered around nearly 100 villages. The major settlements in the district are Utete, the district headquarters and Ikwiriri, Kibiti, Bungu, and Jaribu mpakani, all located along the above-mentioned highway (Fig. 1). The dominant economic activities in the area are smallholder farming (largely conducted along the river valley), carpentry, artisanal fishing, retailing, and of late, animal keeping. The main crops grown in the area are cassava, rice, maize, fruits, vegetables, cashew nuts, and coconuts.

The district is part of hot, humid, coastal plain with varying tropical climatic conditions. It normally gets rain twice a year; the short season, which is more uncertain, is September and October and the longest rainfall happens from February to May. The river gets most of its water during the long rainfall which normally swells and floods a large area of the valley and lasts up to August and September, which is the period that is most favourable for mosquito breeding and when malaria transmission is most intense.

In terms of epidemiologic and health systems studies, the district is famous for the Demographic and Health Systems Surveillance Systems Site (HDSS). At the time preceding this study, Ifakara Health Institute was hosting one of its two HDSS sites in the district. The other one was Ifakara HDSS site in Kilombero district, Morogoro region. Rufiji HDSS activities were conducted in 6 contagious wards composed of 31 villages to the north of Rufiji River, covering an area of 1813 sq km. The site was staffed by a network of 52 paid field workers and supervisors connected to a team of 118 voluntary informants. It created and maintained a database of all households in the villages and their respective residents who were continuously monitored for vital demographic and health events such as birth, migration and death. Cash flow challenges triggered the suspension of the site activities a short time before the start of this study.

Malaria is the key disease in the district. Its transmission is still endemic. It is most common during and after the period of long rain. It is responsible for the majority of out-patient health facility attendance. Young children and pregnant women are population groups at highest risk of transmission [14]. However, the latest progress in its control has extended the burden to school aged children [10]. Other important ones are waterborne disease, soil-transmitted helminths and lower tract respiratory infections, including TB and AIDs. Health care delivery in the district is dominated by 2 hospitals, 5 health centres and 48 dispensaries. They are predominantly owned by the Government. There are several privately owned and operated retail drug outlets authorized by the Government, known as Accredited Drug Dispensing Outlets (ADDOs) which are commonly concentrated in places with relatively large population centres. They are important for increasing access to some key interventions especially for malaria. Malaria control and delivery strategies depend on same interventions provided by national policy [4].

### Study design and procedures

Data were collected using cross-sectional household survey from September to December 2015 and January 2016. Field activities were conducted in 2 phases.

#### Phase 1

The expansion of project activities to areas beyond the HDSS villages forced the team to conduct a census of households and people in the new area before implementation of the survey. The exercise enabled the study to compile a database for sampling selection. The sampling frame was already available in villages covered by HDSS site. The HDSS site wards that were included in the study were Ikwiriri, Mgomba, Umwe, Kibiti, and Bungu. A new non-HDSS ward was added to the study: Chumbi.

#### Phase II

Using the database from both the HDSS and the new enumeration, the survey randomly sampled 2000 households from the 6 wards with an estimated 10,000 members. Two modules of questionnaire were prepared. Module 1 was a questionnaire that collected household information, such as household characteristics and asset ownership. This form was addressed to the head of a household and if he was not available, another senior household member was interviewed. The second module targeted every member of the selected household. Once the household was selected, every member of the selected household was interviewed. As for children aged 16 and below, their parents and caretakers consented and were interviewed on their behalf. A total of 9522 individuals, equivalent to 95.5% of the target population, were interviewed. However, only 7056 individuals, equivalent to 73.5%, were pricked for blood collection. Other people were not pricked because they only accepted to participate in the interviews without consenting to invasive procedures necessary for blood collection. Among the main data collected from the interviews were household asset ownership and household characteristics. They were used to generate household socio-economic status.

Blood drops which were collected were stained on glass slides and stained with Giemsa. They were read by trained microscopists using standard procedures for preparation, interpretation and reporting. Both *P. falciparum* and non-falciparum asexual parasites and gametocytes were identified, but over 98% of malaria infections in these areas were due to *P. falciparum* and prevalence of non-falciparum infection is not reported. Asexual parasites were quantified by counting number of parasites per 200 white blood cells. Parasite density was estimated by assuming a count of 8000 white blood cells per μl of blood. Five per cent of slides read by each microscopist were read again by a senior laboratory technician for quality control; discordant readings were consistently less than 14%.

All two-form modules were prepared and pre-tested in Swahili. They were then installed in tablet computers. The data were recorded in these tablets. Members of the field team were recruited from laid-off HDSS staff. They were familiar with the villages and the households that were collected; because they had worked in this kind of study before using tablets computers, it was easier for them to be trained.

### Data analysis

Data sets were transferred into STATA version 10 software (Stata Corp, College Station, TX, USA) for merging, cleaning and performing analyses. To account for unequal probability of selection, all results were weighted (weight = 1/probability of selection) and were adjusted for clustering with households as the primary sampling unit. The analysis were done with svy: command in STATA. The main outcome measure was the proportion of observed participants with malaria parasites for the whole study. The prevalence was then compared by sex, age group and socio-economic status (SES). SES was estimated using the scores calculated from the household characteristics and asset ownership that were collected from the study. It was generated using principal component analysis (PCA). The prevalence was also analysed for each ward involved in the study. The corresponding distribution of parasitaemia from each ward and sex, age group and SES were also analysed. Comparison of these outcomes within and between wards and the stated risk factors was made using Chi square test. Multivariable logistic regression was used to assess the importance of the selected risk factors for malaria parasitaemia in the study. The Concentration Index formula by Kakwani et al. [5] as adapted in STATA was used to generate concentration indices and the respective confidence intervals from the study to identify existence of socio-economic inequality in malaria parasite prevalence in the study area. The concentration curves were created using MS Excel version 10.

## Results

Table 1 displays respondents that participated in the study and their distribution by basic socio-economic characteristics. Six wards were involved in the study. However, because of many similarities between Ikwiriri, Umwe and Mgomba wards, as they are all and situated at one contagious location, the analysis pooled them as one ward and designated them as Ikwiriri. Ikwiriri will carry the findings for the 3 wards throughout this paper.

**Table 1:** Observed sample from each selected ward and its distribution by a sex, age group and socio-economic status

Ward	N = 9552	Gender		Age groups			Socio-economic status				
		Male n (%)	Female n (%)	< 5 n (%)	5–15 n (%)	> 15 n (%)	Poorest n (%)	Second n (%)	Third n (%)	Fourth n (%)	Least poor n (%)
Ikwiriri	2595	1172 (45.2)	1423 (54.8)	452 (17.4)	792 (30.5)	1351 (52.1)	300 (11.6)	510 (19.7)	531 (20.5)	508 (19.6)	746 (28.8)
Kibiti	2568	1127 (44.0)	1441 (56.0)	479 (18.7)	823 (32.1)	1266 (49.3)	491 (19.1)	394 (15.3)	544 (21.2)	427 (16.6)	712 (27.7)
Bungu	2303	1045 (45.4)	1258 (54.6)	433 (18.8)	779 (33.8)	1091 (47.4)	347 (15.1)	567 (24.6)	569 (24.7)	557 (24.2)	263 (11.4)
Chumbi	2086	1001 (48.0)	1085 (52.0)	396 (19.0)	633 (30.4)	1057 (50.7)	773 (37.1)	445 (21.3)	286 (13.7)	393 (18.8)	189 (9.1)

Table 2 displays malaria parasite prevalence observed from the study and its magnitude from each of the 4 wards. It also shows the distribution of the burden by gender, age group and SES. The overall observed prevalence was 13.0%. The highest prevalence was recorded from Chumbi at 18.4%, which was 41% above the district-wide average. The lowest burden (5.6%) was observed from Ikwiriri: 43% below that of the district. According to Table 2, males were in general significantly more parasitaemic than females (p = 0.000). The burden was consistently higher for school-aged children, 5–15 years compared to under-fives (p = 0.000). Consistent with many other observations, the findings from this study suggest that parasite prevalence was the lowest among the relatively richest individuals than among the poorest (p = 0.000) (Table 2).

**Table 2:** Observed parasitaemia in Rufiji district and its distribution from each selected ward across sex, age group and socio-economic status

Ward	n = 915 (13.0)	Gender		Age group			Socio-economic status				
		Male n = 438 (15.2)	Female n = 477 (11.6)	< 5n = 214 (14.8)	5–15 n = 468 (21.1)	> 15 n = 233 (6.9)	Poorest n = 273 (19.6)	Second n = 198 (14.2)	Third n = 189 (13.7)	Fourth n = 168 (11.8)	Least poor n = 87 (6.1)
Ikwiriri	106 (5.6)	49 (6.4)	57 (5.1)	22 (5.9)	54 (9.4)	30 (3.2)	19 (9.6)	24 (6.1)	24 (6.5)	19 (5.0)	20 (3.7)
Kibiti	241 (12.8)	115 (15.2)	126 (11.2)	64 (15.9)	132 (22.6)	45 (5.0)	84 (21.6)	49 (16.6)	49 (13.4)	28 (9.0)	31 (6.0)
Bungu	290 (16.7)	146 (20.5)	144 (14.1)	62 (17.2)	144 (24.1)	84 (10.8)	57 (21.3)	79 (19.0)	71 (16.3)	68 (16.4)	15 (7.2)
Chumbi	278 (18.4)	128 (18.7)	150 (18.1)	66 (21.6)	138 (29.7)	74 (9.9)	113 (20.7)	47 (15.2)	45 (21.2)	52 (17.2)	21 (14.5)

Overall males p = 0.000

Kibiti sex p = 0.019

Bungu sex p = 0.0001

5–15 group p = 0.000

Poorest p = 0.000

Table 3 presents results generated from multivariate analysis that associates malaria and gender, age group, SES and ITN use. Unlike in univariate analysis as presented in Table 2, where males were more parasitaemic than females (p = 0.000), it is shown in Table 3 that gender had statistically significant relationship with malaria parasitaemia only from Bungu where males were likely to be 40% more parasitaemic than females (95% CI 1.1–1.8. It is similarly shown from the table that people above 15 years and above were less likely by 60% to be infected by malaria parasites compared to under-fives (95% CI 0.3–0.5). The observation was consistent in all of the wards involved in the study. School-aged children (5–15 years) were at higher risk of malaria transmission than under-fives (see Table 3). However, by comparing the two variables according to the wards that the respondents came from, observations were statistically significant only from Kibiti and Bungu.

**Table 3:** Factors related to parasitaemia in Rufiji district by wards

	Districtwide			Ikwiriri			Kibiti			Bungu			Chumbi		
	OR	95% CI	p value	OR	95% CI	p value	OR	95% CI	p value	OR	95% CI	p value	OR	95% CI	p value
Gender															
Female	Baseline														
Male	1.1	1.0–1.3	0.130	1.1	0.8–1.7	0.500	1.1	0.8–1.5	0.580	1.4	1.1–1.8	0.010	0.9	0.7–1.2	0.572
Age group															
< 5	Baseline														
5–15	1.4	1.2–1.8	0.000	1.6	1.0–2.7	0.056	1.5	1.03–2.1	0.036	1.5	1.1–2.2	0.018	1.4	0.9–2.1	0.096
> 15	0.4	0.3–0.5	0.000	0.5	0.3–1.0	0.045	0.3	0.1–0.5	0.000	0.6	0.4–0.9	0.018	0.4	0.3–0.6	0.000
Socio-economic status															
Poorest	Baseline														
Second	0.7	0.5–0.9	0.007	0.6	0.3–1.4	0.273	0.7	0.4–1.2	0.179	0.8	0.5–1.4	0.474	0.7	0.4–1.2	0.164
Third	0.6	0.5–0.8	0.005	0.7	0.3–1.6	0.395	0.6	0.3–1.0	0.040	0.6	0.4–1.1	0.097	1.1	0.7–1.7	0.725
Fourth	0.5	0.4–0.7	0.000	0.6	0.2–1.3	0.196	0.4	0.2–0.6	0.001	0.7	0.4–1.3	0.248	0.8	0.4–1.6	0.388
Least poor	0.3	0.2–0.4	0.000	0.4	0.2–1.0	0.043	0.3	0.2–0.5	0.000	0.3	0.1–0.7	0.003	0.8	0.4–1.6	0.535
Fever presence															
No	Baseline														
Yes	2.4	1.9–2.9	0.000	2.1	1.2–3.7	0.015	3.2	2.1–4.8	0.000	1.8	1.2–2.8	0.009	1.5	1.1–2.1	0.012
ITN use															
No	Baseline														
Yes	0.6	0.5–0.7	0.002	0.6	0.4–0.9	0.045	0.6	0.4–0.8	0.001	0.6	0.4–0.8	0.004	0.8	0.6–1.1	0.159

In terms of SES, the wealthiest individuals in the district were observed to be less vulnerable to malaria parasites compared to the poorest (95% CI 0.2–0.4). These district-wide results were comparable to those from Kibiti and Bungu where the least poor were less parasitaemic than the poorest by 70%.

The results suggest an overall 60% reduction in malaria parasitaemia in people sleeping under an LLIN compared to people who do not use them. Protection provided to LLIN users was maintained in every ward selected for the study, except from Chumbi. Fever prevalence also predicted parasitaemia from the study. People that reported fever 14 days preceding interview were twice as parasitaemic as others who did not report the condition.

Table 4 reports LLIN use for the district and from each selected ward and its distribution by gender, age group and SES. Overall, LLIN use from the district was 58%. Females reported higher LLIN use than males (p = 0.000) and children younger than 5 years reported the highest LLIN use of all other age groups (p < 0.05), and school-aged children had the smallest proportion of LLIN use (p = 0.000). The highest LLIN coverage was observed from Ikwiriri and the lowest was observed from Bungu. Females consistently reported higher LLIN coverage than males in all wards (p < 0.05). School-aged children were least protected in every ward. In terms of wealth quintiles, the least poor segment of the population in every community enjoyed higher ITN coverage than the poorest (p < 0.05).

**Table 4:** Insecticide treated net use in Rufiji district and its distribution from each selected ward across sex, age group and socio-economic status

Ward	n = 5285 (57.5)	Gender		Age group			Socio-economic status				
		Male n = 2241 (54.2)	Female n = 3044 (60.2)	< 5 n = 1095 (63.6)	5–15 n = 1427 (48.4)	> 15 n = 2763 (61.1)	Poorest n = 543 (39.2)	Second n = 691 (51.2)	Third n = 772 (55.7)	Fourth n = 887 (60.0)	Least poor n = 1080 (80.1)
Ikwiriri	1728 (69.6)	727 (66.0)	1001 (72.4)	341 (76.3)	490 (64.1)	897 (70.6)	67 (34.5)	233 (62.8)	257 (71.2)	294 (76.8)	434 (83.0)
Kibiti	1289 (52.2)	522 (48.7)	767 (55.0)	279 (60)	341 (42.5)	669 (55.7)	579 (57.8)	92 (34.0)	170 (49.6)	179 (58.5)	379 (76.9)
Bungu	1030 (46.3)	441 (44.1)	589 (48.0)	215 (51.1)	287 (37.4)	528 (50.9)	89 (34.5)	178 (41.8)	179 (40.3)	224 (56.3)	160 (76.6)
Chumbi	1238 (61.4)	551 (57.3)	687 (65.2)	260 (66.7)	309 (50.4)	669 (66.0)	249 (45.7)	188 (66.9)	166 (70.0)	190 (68.6)	107 (87.0)

Overall sex p = 0.000

Ikwiriri sex p = 0.001

Kibiti sex p = 0.002

Chumbi sex p = 0.003

**Fig. 2:**
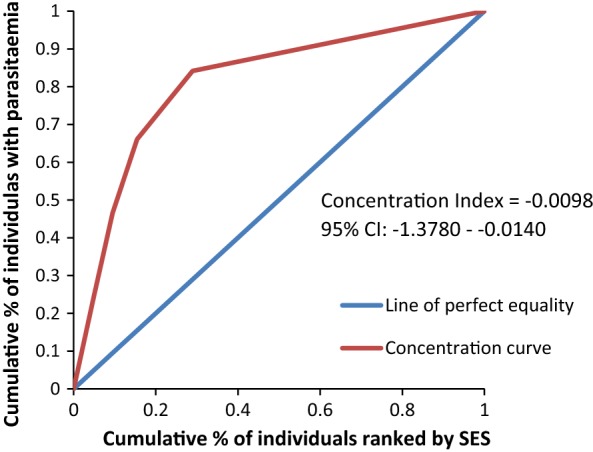
Socio-economic inequality in malaria parasitaemia as generated from the national malaria survey 2015–2016

**Fig. 3:**
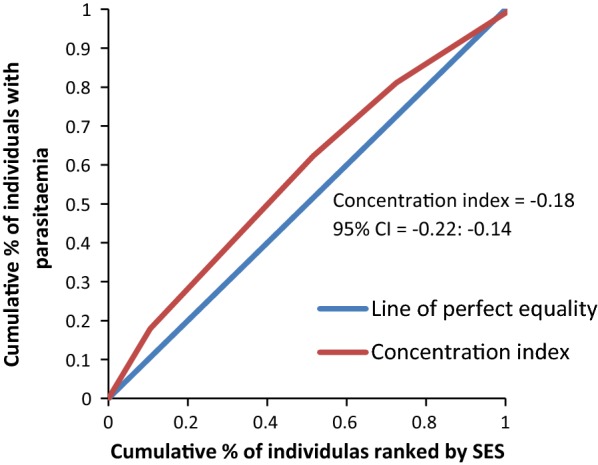
Socio-economic inequality in malaria parasitaemia in Rufiji generated from the study survey

**Fig. 4:**
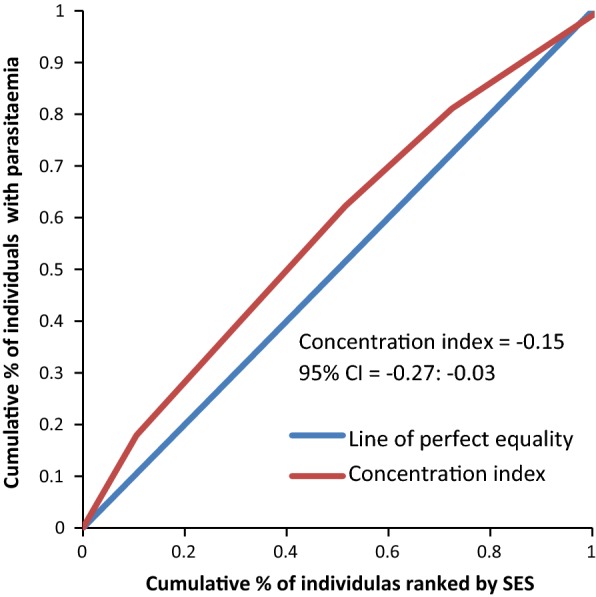
Socio-economic inequality in malaria parasitaemia in Ikwiriri generated from the study

**Fig. 5:**
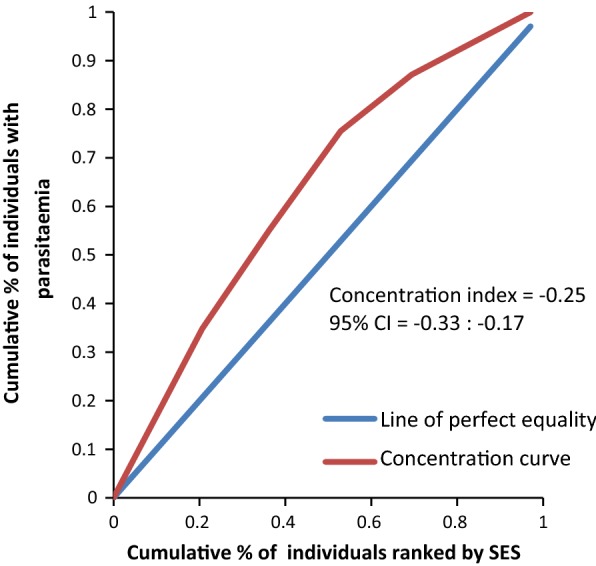
Socio-economic inequality in malaria parasitaemia in Kibiti generated from the study

**Fig. 6:**
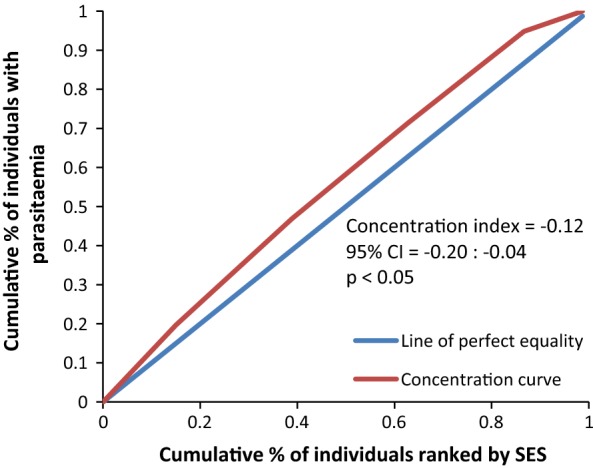
Socio-economic inequality in malaria parasitaemia in Bungu generated from the study

**Fig. 7:**
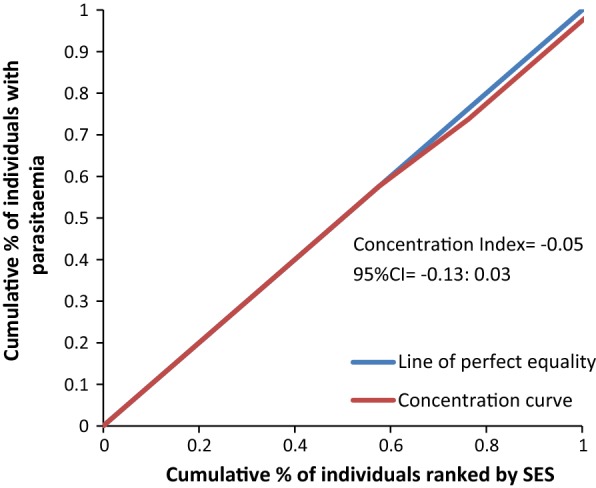
Socio-economic inequality in malaria parasitaemia in Chumbi generated from the study

The concentration curves plotted using data from the national survey as depicted in Fig. 2 lie above the line of perfect equality, suggesting the existence of disproportionate inequality in malaria parasite prevalence against the poorest population in the country. This is supported by the negative CIX (− 0.0098) that corroborates the curve. However, since the index is so small it suggests that the concern is not so serious and it can easily be fixed. Figure 3 represents the overall situation from the study and follows the national pattern. In contrast, the CIX is larger away from 0 showing a bigger magnitude of parasitaemia inequality afflicting the poorest population in Rufiji district *versus* the country. The overall pattern from the study was repeated in all wards (Figs. 4, 5, 6) involved in the study, except Chumbi (Fig. 7). The CIX from all wards showing concentration curves above the line of equality suggests inequality in malaria burden that is more severe than the national average.

**Fig. 8:**
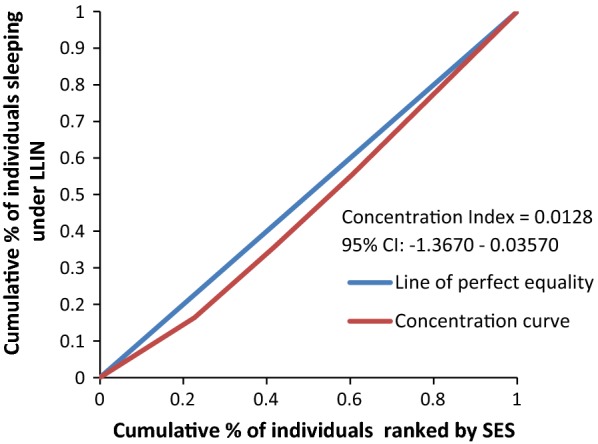
Socio-economic inequality in LLIN use generated from the national survey 2015–2016

**Fig. 9:**
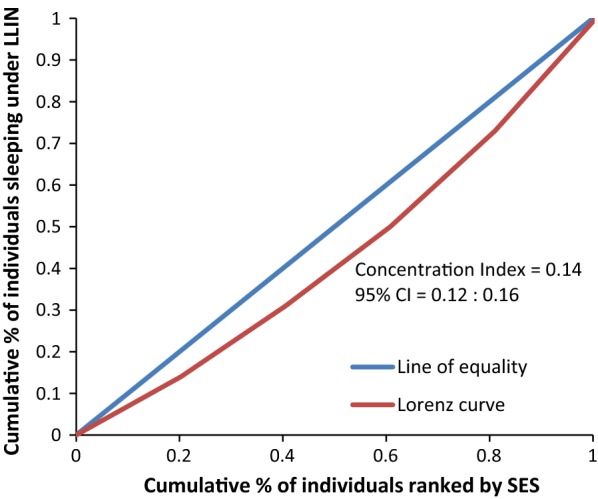
Socio-economic inequality in LLIN use in Rufiji district generated from the study

**Fig. 10:**
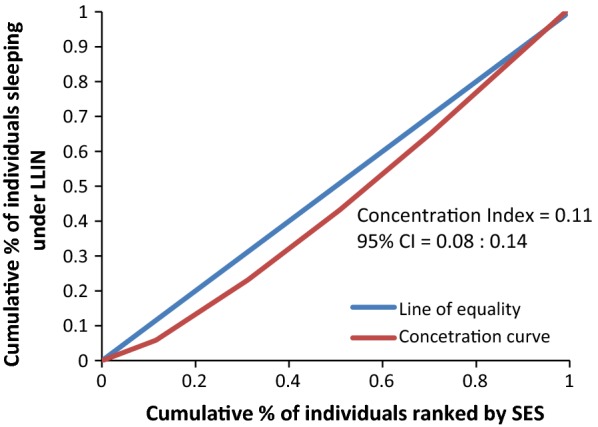
Socio-economic inequality in LLIN use in Ikwiriri generated from the study

**Fig. 11:**
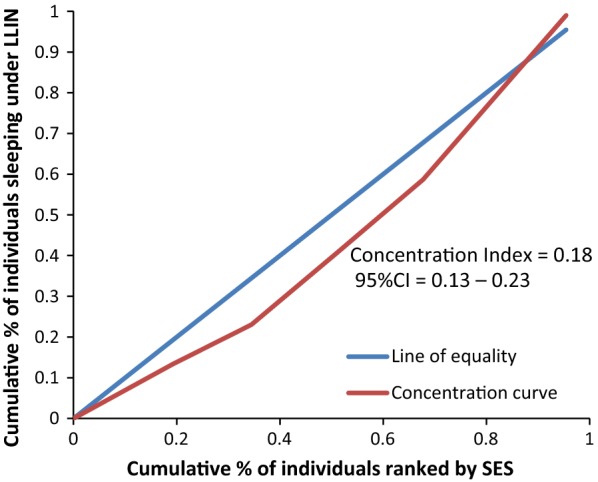
Socio-economic inequality in LLIN use in Kibiti generated from the study

**Fig. 12:**
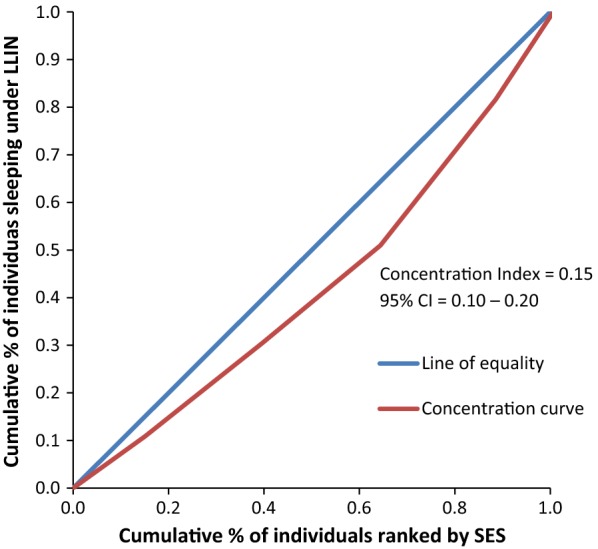
Socio-economic inequality in LLIN use in Bungu generated from the study

**Fig. 13:**
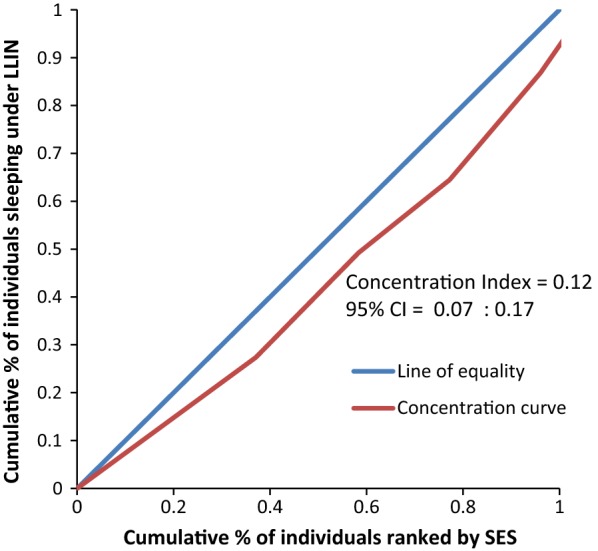
Socio-economic inequality in LLIN use in Chumbi generated from the study

Figure 8 rules out the existence of average meaningful socio-economic inequality in LLIN use in the country. The fact that all curves lie below the diagonal supported by positive values in CIX with confidence intervals to the right of 0 from Figs. 9, 10, 11, 12, 13 demonstrates that national data are hiding the true condition of LLIN use inequality in some narrow geographical settings. The figures reveal the weakness of the aggregate data presented in the national survey in evaluating equality of coverage in malaria interventions.

Figures 4, 5, 6, 7 demonstrates concentration curves and indices that depict equal socio-economic inequality based on malaria parasitaemia and ITN coverage from the study. Figure 2 shows the concentration curve lying above the perfect line of equality, which can be interpreted as higher exposure to malaria parasite prevalence for the poorest. The overall magnitude of inequality for the district and across the wards is reflected by concentration indices below 0 (− 0.1; 95% CI − 0.21 to 0.13). This disproportionate distribution of parasitaemia discriminating against the poor was statistically significant in the district. Concentration curves presented in Figs. 2, 3, 4, 5, 6 lie below the diagonal generated from data observed from the study, and supports other findings from the national survey. The concentration curve from this graph was above 0 (0.13; 95% CI 0.11–0.15).

## Discussion

This is the first study demonstrating malaria parasite prevalence in a narrow geographic setting in Tanzania since the TDHS–MIS 2015–2016 [1]. The average parasitaemia observed from the study was larger than the national average. National malaria prevalence as measured using microscopy reported for 2016 was 6%. However, the national malaria data were limited to under-fives and disaggregated down to only regional level. Pwani, a region to which Rufiji district belongs, reported malaria parasite prevalence for under-fives of only 5.8%. Under-five parasitaemia from this study was 14.8%. This is a large variation between the national and district level data that demonstrates that even if the overall country’s malaria burden is in decline as in some countries in Africa [6], the problem is shifting towards certain geographical locations in the same country. This variation was reported even between various regional divisions. Some regions in the central, northeastern and southern highland zones had parasite prevalence lower than 1% while in regions around Lake Victoria, such as Geita, parasitaemia was 17.7% [1].

It is here been presented this kind of variation in parasitaemia between the 4 sub-district divisions that were selected for the study. While an average parasitaemia was 13%, Ikwiriri showed parasite prevalence lower than 6% and Chumbi, which was the most parasitaemic ward, had a prevalence of 18%. Variation was expanded to include some other factors that were in many other studies associated with malaria transmission. It has been reported that parasitaemia was more common for school-aged children compared to other groups, including under-fives who, together with pregnant women, are still considered to be at highest risk of malaria [6]. It is because of this prevailing knowledge that most malaria-evaluating studies, including malaria indicator surveys, are concentrating on under-fives [1, 7, 8]. The changing malaria risks that are expanding or shifting to older children have been reported in several other studies [9, 10]. Unequal malaria distribution based on SES has also been highlighted in this study. The disease is most common among poorest groups in the community. Poverty as a risk exposure to malaria has been reported elsewhere [8, 11–14]. The lowest parasitaemia observed from the study was in a ward with largest proportion of least poor, and possessed the largest features of an urban settlement. This observation is consistent with other studies on malaria epidemiology in Africa [15, 16]. It can, therefore, be deduced that malaria is a disease of poverty and is more concentrated on the fringes of a population. Additional progress on control and subsequent elimination requires efforts that will concentrate on the poorer populations in poorer settlements.

Long-lasting insecticidal net use is scaled up as a control tool for vectors responsible for malaria transmission. Many and different programmes have been implemented in Tanzania and in many other places that are at risk of malaria [17–19]. Effective LLIN coverage can provide both personal and community protection against malaria [20–22] even though its validity is counter-indicated by an increasing population of *An*. *arabiensis*, a sister species of *An*. *gambiae* complex which poses a major setback to existing vector control strategies [23]. It has been shown in a number of studies that LLIN roll out is responsible for the decline of malaria burden observed in many places [24–26]. Three major strategies used for rolling out LLINs in Tanzania are a universal coverage campaign (in 2010–2011), private sector distribution and School Net Programme [1, 17]. From a 2015–2016 survey, an average 50.7% was collectively achieved for the country [1]. This study has reported higher average LLIN use than national figures. It has been shown that malaria prevalence was not homogenous across the study. With the exception of one location, whose case will be discussed in a separate paragraph, malaria parasite prevalence was inversely related to LLIN use. The burden was largest where LLIN use was the lowest and it was the lowest where LLIN use was the highest. This observation cannot and is not supported at every place because malaria transmission functions in a multitude of conditions and its control is multifactorial [8, 10]. A number of studies have demonstrated a significant decline in malaria that was not explained in terms of high LLIN coverage [8, 10]. Achieving effective coverage, under certain conditions, leads to lower malaria burden. However, there are interventions, including changes in environmental conditions and secular trend, which can bring the malaria burden down independent of LLIN coverage [27, 28]. For example, a TDHS–MIS report showed that not all regions reported with highest malaria parasite prevalence had the lowest LLIN coverage [1]. Indeed, the Government and its partners strive to achieve LLIN coverage over 80% by 2020 [29]. This goal has not yet been achieved as the current coverage is 49% [1].

As for larger parasite burden observed from school-aged children *versus* under-fives and other population groups, LLIN follow the same pattern. This age category possessed the lowest LLIN coverage in the study. This observation is supported by results from the TDHS–MIS that suggest LLIN use was highest for under-fives and among people living in urban areas [1]. Females were also associated with higher LLIN use than males. The variation can be associated with LLIN distribution programmes targeting under-fives and pregnant women as the existing knowledge singles them out as biologically most vulnerable to malaria transmission [6]. However, it is important for new malaria control plans to be aware of this new reality, as success in sustaining gains and accelerating progress towards end goals in the fight against malaria will depend on clearing parasitaemia from everyone. This is so important for the prevention of onward transmission. Programmes intended to improve malaria interventions for the currently recognized vulnerable groups should be modified to include other groups observed with highest parasitaemia.

It has been stated that a high malaria parasitaemia observed from Chumbi ward presents a paradox for a pattern that shows an association between high LLIN use and low malaria parasitaemia and improved SES and high LLIN coverage. The ward was characterized by the lowest proportion of residents in the highest SES but had higher LLIN use next to Ikwiriri. However, being in the top league of LLIN users was not reflected in parasitaemic status which was the highest of all wards in the study. Various studies have reported that massive roll out of existing malaria vector control interventions have a devastating impact on mosquito species *An. gambiae* sensu stricto that formerly played a leading role as a malaria vector in Africa [23, 30, 31]. Implementation of LLINs alone or in combination with IRS has successfully reduced the population of this mosquito species bringing many malaria-endemic countries close to goals of malaria control. However, the emergence of *An. arabiensis*, a sister species of *An. gambiae* complex, which can survive on both bovine and human blood, poses a new challenge to eliminating malaria. Their susceptibility to control has been attenuated by their ability to feed outdoor whether at dusk or dawn on both humans and animals, in the case of indoor human protection with existing vector control interventions [33]. Being zoophagic, it has been shown that they are more abundant in areas with large animal populations as was the case in Chumbi ward where domestication of animals was one of its major economic activities [23, 32]. A substantial number of residents in the area are pastoralists who tend to spend prolonged hours with their animals which likely exposes them to opportunistic malaria vectors, and which have exhibited insecticide avoidance behaviour. Malaria protection potential of LLINs, whose usage in Chumbi had surpassed several other locations in the study, could hardly be optimized due to conditions discussed. This paradox was not limited to this study area. TDHS–MIS also shows the highest malaria parasite prevalence in regions that are predominantly pastoralists [1]. However, LLIN coverage was higher than in some other regions, reporting relatively low parasitaemia. This could vindicate entomological models demonstrating decreased protection potential of conventional vector control measures in the face of changing dynamics of vector composition [23, 33, 34].

Many studies have identified inequality as an important barrier to achieving universal coverage of malaria control interventions that is necessary for achieving total control of the disease [35, 36]. The findings from the study have shown various forms of inequality. The most important one that has consumed enormous resources to fix was inequality based on SES. Logistic regression model presented in results section supported by concentration curves and concentration indices has demonstrated the presence and significant magnitude of wealth-based inequality in parasitaemia and LLIN use. All concentration curves generated using malaria parasitaemia data are hovering above the line of perfect equality. All CIX lie to the left of 0 and can be interpreted that malaria is concentrated in the poorest population. Concentration curve from national malaria data is no different from that observed in this study. Equally important, several evaluation studies conducted in Africa have demonstrated that poverty is a risk factor for malaria [13, 37, 38]. Poor access to malaria control interventions has often been cited as a valid explanation for this. Of all interventions in place, the study paid attention to LLINs only. It showed the lowest LLIN use among the poorest study participants as reflected in concentration curves lying below the diagonal.

The question to ask is, what did happen to the LLIN universal campaign aimed at addressing poverty as an obstacle to access? Is the investment worth pursuing? TDHS–MIS and other studies following up sources of nets providing coverage to different socio-economic groups found that LLINs from mass campaigns were responsible for the largest proportion of nets used by the poorest population [39]. These nets were commonly reported from rural areas. Conversely, private sector distribution was responsible for the majority of nets used by the least poor population [22]. This was commonly reported in urban areas. A source of LLINs in this type of setting is a function of SES that trickles down to urban and rural areas. It is evident that mass distribution campaigns are universal and periodic while the private sector is continuous but only active in urban areas. Time intervals between mass campaigns is longer than LLIN lifespan which can account for a variation in reported LLIN use between the quintiles and between rural and urban areas. In addition, publicly funded mechanisms intended for sustaining high level LLIN coverage for vulnerable populations is delivered in formal sector sources whose access in rural areas and to poor populations in urban areas is narrow. Consequently, it is largely beneficial to relatively better off people concentrated in urban areas.

## Conclusion

The study has shown malaria parasite prevalence based on microscopic examination higher than the national average. It has shown substantial variations in parasitaemia between sub-district locations. This condition gives an impression that malaria control efforts in the study area have yielded varying impact on malaria burden. It appears that the burden is concentrated in areas and among groups with limited access to control interventions. It suggests that implementation of malaria control activities are favourable in easy-to-reach areas and to groups able to afford the costs involved in accessing interventions targeted by publicly subsidized programmes. These are areas that are characterized by relatively good healthcare services that provide better access to such key malaria control interventions as LLINs and subsidized healthcare facilities. Population groups with economic strength can afford the high prices charged for malaria control interventions. The fact that LLIN use was highest in population centres, among under-fives, women, and the highest socio-economic quintile provides reasonable evidence to this conclusion.

It is evident that inaction is not an option for national malaria control programmes striving for progress in malaria control and malaria elimination. For success in malaria control in some areas and in some population groups, while leaving out peripheral areas and other marginal groups, will undo gains made and take the country back to where it was over a decade ago. It is important to identify and implement strategies that have worked elsewhere. Programmes that have worked to increase LLIN coverage for under-fives and pregnant women can be expanded to include school-aged children, adult males and non-pregnant females. The study has overall provided a broad picture of populations based parasite burden in Tanzania at sub-national level. However, its strength may have been in some ways been affected by the limitation of cross-sectional surveys. Data were collected in December and January, which are the peak season for malaria transmission in the area, but can hardly represent all of the remaining months of the year. Members of some sampled households could not be interviewed because they were not present at their homesteads during the time of the study. Their absence could have biased the results in some ways. Sample selection was based on households and therefore some population groups might have disproportionate representation and this would also have swayed the results.
